# Demonstration of the Broad-Spectrum *In Vitro* Activity of Finafloxacin against Pathogens of Biodefense Interest

**DOI:** 10.1128/AAC.01470-19

**Published:** 2019-11-21

**Authors:** Kay B. Barnes, Steven D. Zumbrun, Stephanie A. Halasohoris, Purvi D. Desai, Lynda L. Miller, Mark I. Richards, Paul Russell, Christine Bentley, Sarah V. Harding

**Affiliations:** a Defence Science and Technology Laboratory, Porton Down, Salisbury, United Kingdom; b U.S. Army Medical Research Institute of Infectious Diseases, Fort Detrick, Maryland, USA; c MerLion Pharmaceuticals, Berlin, Germany

**Keywords:** finafloxacin, *in vitro* activity, acidic pH, biothreat pathogens, acid environments, biodefense, *in vitro*

## Abstract

This study investigated the *in vitro* activity of finafloxacin against bacterial strain panels of the biodefense pathogens. Broth microdilution assays were performed at neutral and acidic pH to determine the effectiveness of the antibiotics under conditions typical of an intracellular environment. In all instances, finafloxacin demonstrated superior activity at low pH. These results highlight the importance of evaluating antimicrobial efficacy under conditions relevant to those encountered *in vivo*.

## Abstract (precis)

This study investigated the *in vitro* activity of finafloxacin against bacterial strain panels of the biodefense pathogens. Broth microdilution assays were performed at neutral and acidic pH to determine the effectiveness of the antibiotics under conditions typical of an intracellular environment. In all instances, finafloxacin demonstrated superior activity at low pH.

## TEXT

Antimicrobial resistance is an evolving issue, and new therapeutics are needed to treat infections caused by the pathogens of biodefense interest and those that are considered to be of public health concern. It is important that new antimicrobials are evaluated under conditions that model those encountered within the environment of a host, including the low-pH environment of the cell (the phagolysosome) that is particularly relevant to intracellular pathogens and infected body sites. It has been shown previously that the activity of certain classes of antibiotics (including fluoroquinolones) can be affected by a reduction in pH (1–4). Finafloxacin is a fluoroquinolone derivative with an 8-cyano substituent and 7-pyrrolo-oxazinyl moiety that is being developed for the treatment of urinary tract infections in hospitalized patients (5, 6). This modification has conferred activity in low-pH environments, which has resulted in superior *in vitro* activity against a range of organisms, including Staphylococcus aureus and Acinetobacter baumannii (7, 8).

The availability of formulations of finafloxacin that can be delivered orally and systemically makes finafloxacin a worthy alternative for the treatment of a range of infections. In addition to good safety and efficacy data obtained in patients suffering from complicated urinary tract infections and pyelonephritis, previous studies have also demonstrated efficacy against the biothreat agents Burkholderia pseudomallei and Francisella tularensis *in vitro* and *in vivo* (6, 9–11). The aim of this study was to further evaluate the *in vitro* activity of finafloxacin against larger strain panels of biodefense pathogens.

Antibiotic susceptibility was determined at pH 5 and pH 7 for B. pseudomallei (*n* = 10), Burkholderia mallei (*n* = 10), F. tularensis (*n* = 10), Bacillus anthracis (*n* = 10), and Yersinia pestis (*n* = 10), held at the U.S. Army Medical Research Institute of Infectious Diseases (USAMRIID) (Table 1). In addition, a B. pseudomallei strain panel (*n* = 11) provided by the Biomedical Advanced Research and Development Authority (BARDA) was screened (Table 1) (12). These assays were performed under biosafety level 3 (BSL3) conditions. Antibiotic susceptibility was reported as the MIC_50_ or MIC_90_, defined as the lowest concentration of the antibiotic at which the growth of 50% or 90% of the isolates, respectively, were inhibited.

**TABLE 1:** Panel of bacterial strains evaluated

Organism	Strain^*a*^	Origin	Source
B. pseudomallei	316c	Thailand	Human
	E203	Thailand	Unknown
	NCTC4845	Singapore	Monkey
	STW115-2	Thailand	Water
	STW199-2	Thailand	Water
	E8	Thailand NE	Soil
	P52237	Vietnam	Unknown
	WRAIR1188	Malaysia	Human
	K96243	Thailand	Human
	1026b	Thailand	Human
	K96243*	Thailand	Human
	1026b*	Thailand	Human
	HBPUB10134A*	Thailand	Human
	HBPUB10303A*	Thailand	Human
	1106a*	Thailand	Human
	MSHR 305*	Australia	Human
	MSHR 668*	Australia	Human
	MSHR 5855*	Australia	Human
	MSHR 5848*	Australia	Human
	MSHR 5858*	Thailand	Human
	406e*	Thailand	Human
F. tularensis	LVS	Former Soviet Union	Water rat
	OR01-1807	USA	Unknown
	FRAN003	USA	Unknown
	FRAN005	USA	Unknown
	FRAN006	USA	Unknown
	FRAN007	USA	Unknown
	FRAN012	USA	Unknown
	FRAN013	USA	Unknown
	FRAN016	USA	Unknown
	SCHUS4-1	USA	Human
B. anthracis	Vollum1B	USA	Bovine
	Sterne	South Africa	Bovine
	Ames	USA	Bovine
	K1938	Indonesia	Unknown
	K5926	India	Unknown
	K7038	South Korea	Unknown
	SK57	England	Unknown
	K7978	Namibia	Unknown
	Africa33	South Africa	Unknown
	K8091	Norway	Unknown
B. mallei	GB3 (ATCC 120)	UK	Unknown
	GB4	Turkey	Human
	GB5	Hungary	Unknown
	GB6	Turkey	Human
	GB7	Turkey	Unknown
	GB8 (China7)	Burma	Human
	GB9	India	Mule
	GB10	India	Horse
	GB11	China	Horse
	GB12	Hungary	Unknown
Y. pestis	CO92	USA	Human
	C12	USA	Human
	antiqua	Congo	Human
	pestoidesB	Former Soviet Union	Human
	pestoides Fmp1	Former Soviet Union	Human
	Yeo154	Japan	Human
	Angola	Angola	Human
	Java9	Indonesia	Human
	M111(74)	Madagascar	Human
	LaPaz	Bolivia	Human

a Strains with an asterisk belong to the BARDA strain panel. All other strains were obtained from the USAMRIID Unified Culture Collection (UCC), Frederick, MD, USA.

Finafloxacin was supplied by MerLion Pharmaceuticals GmbH, and all other antibiotics were sourced from the U.S. Pharmacopeia, Selleckchem, or Sigma. Broth microdilution assays were performed as detailed by the Clinical and Laboratory Standards Institute (CLSI) (13), with the exception of a medium supplement (2%), IsoVitaleX (Becton, Dickinson), used to support the growth of F. tularensis. The activity of finafloxacin was determined at pH 5 and pH 7 (if the bacterial species was able to be cultured) and the MICs determined.

**FIG 1:**
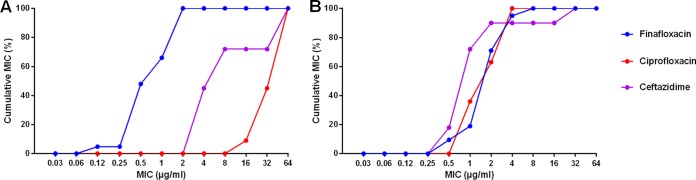
Cumulative MICs determined for a panel of B. pseudomallei strains for finafloxacin (*n* = 21), ciprofloxacin (*n* = 11), and ceftazidime (*n* = 11) at pH 5 (A) and pH 7 (B).

At pH 5, the MICs for B. pseudomallei ranged from 0.12 to 2 μg/ml, 16 to 64 μg/ml, and 4 to 64 μg/ml for finafloxacin, ciprofloxacin (CIP), and ceftazidime (CAZ), respectively, demonstrating the superior *in vitro* activity of finafloxacin at low pH. Although it is difficult to make comparisons between the efficacies of antibiotics simply by MIC, these values are lower than those determined for another fluoroquinolone, CIP, and a component of the treatment for B. pseudomallei infections in humans (CAZ) (Fig. 1A). At neutral pH, finafloxacin demonstrated a level of activity (0.5 to 8 μg/ml) similar to those observed with CIP (1 to 4 μg/ml) and CAZ (0.5 to 32 μg/ml) (Fig. 1B).

A similar trend was observed with the other pathogens of biodefense interest. Finafloxacin had superior activity at pH 5 for B. anthracis, B. mallei, and Y. pestis compared to either CIP or azithromycin (AZM) (an antibiotic used for the treatment of B. mallei infection in humans and as a control in the *in vitro* assays) (Table 2). Unfortunately, only two strains of F. tularensis could be cultured in this low-pH environment; therefore, the MIC_50_ and MIC_90_ at pH 5 could not be determined. The most striking difference was observed for B. mallei. Finafloxacin had 9-fold and 7-fold improved activity over that of AZM against a panel of these strains (MIC_50_, 0.12 μg/ml compared to >64 μg/ml; MIC_90_, 0.5 μg/ml compared to >64 μg/ml) when performed at pH 5 (Table 2). At pH 7, finafloxacin demonstrated activity similar to those of the comparator antibiotics, with MIC_50_ and MIC_90_ of 0.5 μg/ml (at both pHs) for B. mallei and 0.06 μg/ml and 0.12 μg/ml for B. anthracis, respectively (Table 2).

**TABLE 2:** MIC_50_, MIC_90_, and MIC range values determined for panels of the biothreat pathogens

Species	MIC (μg/ml) by pH^*a*^																	
	MIC_50_						MIC_90_						Range					
	pH 5			pH 7			pH 5			pH 7			pH 5			pH 7		
	FIN	CIP	AZM	FIN	CIP	AZM	FIN	CIP	AZM	FIN	CIP	AZM	FIN	CIP	AZM	FIN	CIP	AZM
B. anthracis	≤0.03	0.06	ND	0.06	0.03	ND	≤0.03	0.06	ND	0.12	0.03	ND	≤0.03 to 0.06	0.03 to 0.06	ND	0.06 to 0.12	0.03 to 0.06	ND
B. mallei	0.12	ND	>64	0.5	ND	0.25	0.5	ND	>64	0.5	ND	0.5	≤0.03 to 0.5	ND	4 to >64	≤0.03 to 0.5	ND	0.06 to 0.5
Y. pestis	≤0.03	0.5	ND	≤0.03	0.015	ND	≤0.03	1	ND	0.06	0.03	ND	≤0.03	0.12 to 1	ND	≤0.03 to 0.12	0.008 to 0.03	ND
F. tularensis	ND	ND	ND	≤0.03	0.015	ND	ND	ND	ND	≤0.03	0.03	ND	ND	ND	ND	≤0.03	0.008 to 0.25	ND

a ND, not determined.

The data set detailed in these studies demonstrates that finafloxacin has activity under both acidic and neutral conditions, with enhanced activity of finafloxacin in low-pH environments, where other antibiotics (including ciprofloxacin) have reduced activity. This has been demonstrated for all of the biodefense pathogens of interest and is in agreement with data generated by other groups (7, 8, 10, 11). The improved activity of finafloxacin compared to that of ciprofloxacin (a typical treatment for infections caused by B. anthracis, Y. pestis, and F. tularensis) further highlights the importance of evaluating therapies under conditions considered to be more like those encountered within a host and identifies finafloxacin as a novel broad-spectrum fluoroquinolone that could be used for prophylaxis or treatment following exposure to a range of pathogens.

Of particular interest is the activity of finafloxacin against the Burkholderia species evaluated. It has been demonstrated previously that fluoroquinolones are not effective as treatment for melioidosis in humans mainly due to B. pseudomallei possessing resistance mechanisms, including efflux pumps (14–17). The results detailed in this communication suggest that finafloxacin is not affected by the efflux pumps in B. pseudomallei that confer resistance to other fluoroquinolones, possibly due to the effect of the modified chemical structure (7, 10, 18). The promising data generated for B. mallei suggest that finafloxacin is a potential alternative for the treatment of infection caused by this organism.

Finafloxacin appears to have a wider spectrum of activity than the other fluoroquinolones and has the potential to be used to treat infections caused by all of the biothreat pathogens evaluated (19). It has also been shown to be safe and well tolerated in clinical trials (6). These encouraging *in vitro* findings warrant further investigation of finafloxacin which would determine whether this activity translates into comparable protection against all of these pathogens *in vivo*.
